# Geographic Differences in Preconception Health Indicators Among Ohio Women Who Delivered Live Births, 2019–2021

**DOI:** 10.5888/pcd21.230244

**Published:** 2024-02-08

**Authors:** Natalie DiPietro Mager, Michelle Menegay, Connie Bish, Reena Oza-Frank

**Affiliations:** 1 Ohio Northern University Raabe College of Pharmacy, Ada, Ohio; 2 Ohio Colleges of Medicine Government Resource Center, The Ohio State University Wexner Medical Center, Columbus; 3 Division of Reproductive Health, National Center for Chronic Disease Prevention and Health Promotion, Centers for Disease Control and Prevention, Atlanta, Georgia

## Abstract

To determine whether geographic differences in preconception health indicators exist among Ohio women with live births, we analyzed 9 indicators from the 2019–2021 Ohio Pregnancy Assessment Survey (N = 14,377) by county type. Appalachian women reported lower rates of folic acid intake and higher rates of depression than women in other counties. Appalachian and rural non-Appalachian women most often reported cigarette use. Suburban women reported lower rates of diabetes, hypertension, and unwanted pregnancy than women in other counties. Preconception health differences by residence location suggest a need to customize prevention efforts by region to improve health outcomes, particularly in regions with persistent health disparities.

**Summary**

**What is already known on this topic?**

Monitoring preconception health can help identify strategies to optimize women’s health and birth outcomes; however, reporting these measures at state or national levels may mask potential variation.

**What is added by this report?**

We analyzed data from the 2019–2021 Ohio Pregnancy Assessment Survey by county type and found significant differences for 6 of 9 priority preconception health indicators.

**What are the implications for public health practice?**

Differences in preconception health by residence location emphasize the need for regional customization of prevention efforts to improve health outcomes, particularly in regions with persistent health disparities.

## Objective

The life course perspective recognizes that good preconception health is key to mitigating adverse maternal–fetal outcomes and optimizing women’s biomedical, behavioral, and social health throughout the lifespan (1–5). States with high rates of poor maternal and infant outcomes may find particular benefit from monitoring preconception health to identify strategies to improve birth outcomes (2). Ten priority surveillance indicators were previously identified to assess population-level well-being among reproductive-age women: folic acid intake, normal weight, effective contraceptive use postpartum, heavy alcohol use, smoking, physical activity, depression, diabetes, hypertension, and unwanted pregnancy (2). These indicators are used for benchmarking, monitoring, allocating resources, and developing programs and policies (2). Often, these measures are reported at national or state levels; however, disaggregating and reporting these measures for rural or underserved areas may be necessary to better understand regional needs (2,6). Ohio is a large state with 88 counties encompassing 4 mutually exclusive geographical regions (Appalachian, which spans rural to metropolitan areas; metropolitan; rural non-Appalachian; and suburban) based on population size and county designation by the Appalachian Regional Commission (www.arc.gov) (Figure) (7). Therefore, we sought to determine whether geographic differences in priority preconception health indicators exist among Ohio women who delivered live births.

**Figure:**
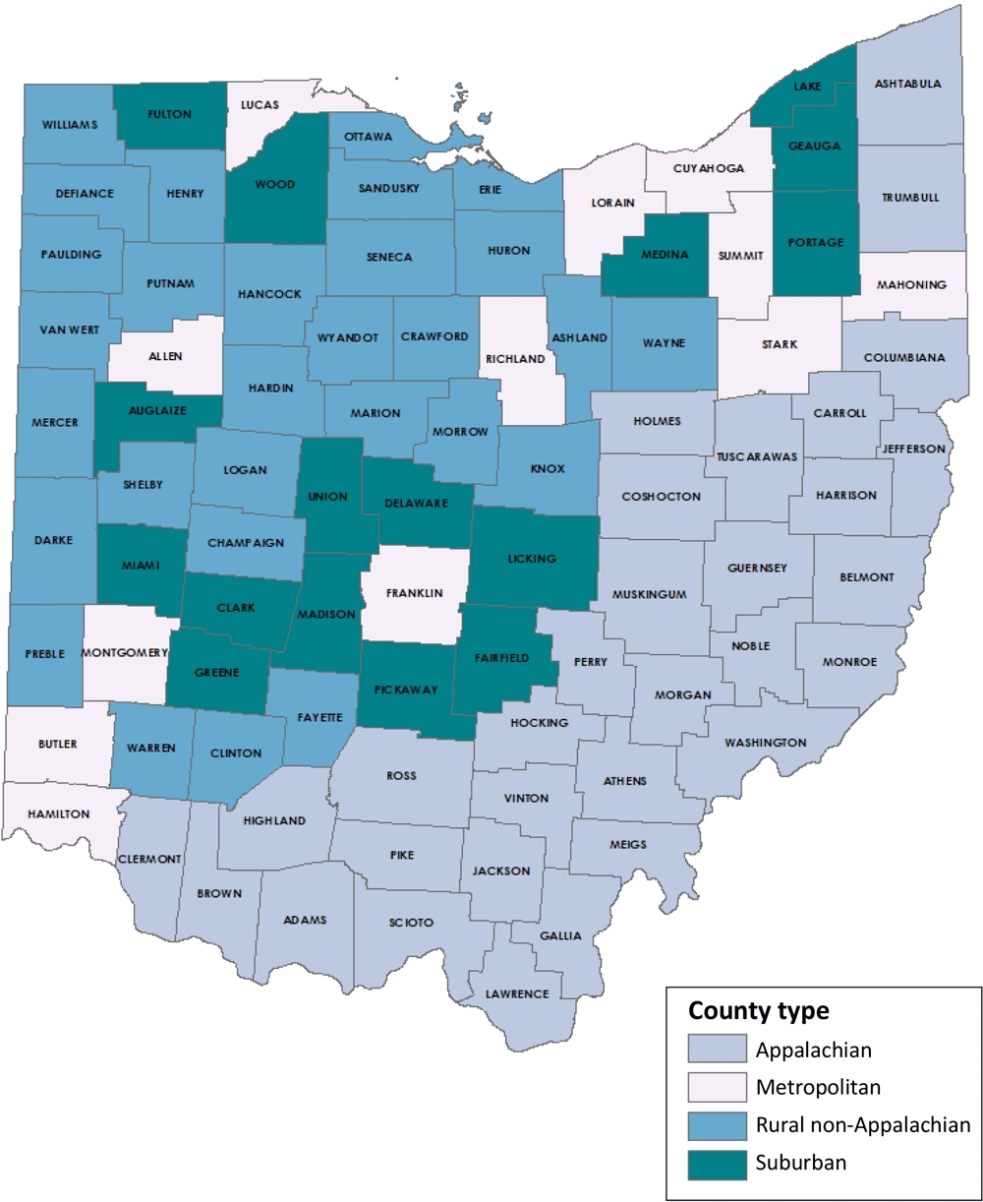
Ohio county types, developed by Ohio Department of Health, Bureau of Vital Statistics based on county population size and county designation by the Appalachian Regional Commission (7).

## Methods

We analyzed 2019–2021 Ohio Pregnancy Assessment Survey (OPAS) data. OPAS is a statewide, population-based, cross-sectional survey that monitors resident women who experienced a live birth (8). Briefly, OPAS collects data from a random, stratified sample of women selected from Ohio’s birth certificate data. Women are invited to participate in OPAS 2 to 6 months after delivery using 1 of 3 survey modes (web, mail, or telephone in either English or Spanish) (8). Completed survey data are then linked to data from the infant birth certificate (8). The OPAS response rates were 40.5% (2019), 38.4% (2020), and 36.1% (2021).

We performed descriptive analyses by using survey weights to estimate weighted prevalence and 95% CIs for 9 of the 10 priority preconception health indicators overall and by county type. Among the 9 priority preconception health indicators, 3 indicate health promotion: 1) folic acid intake (multivitamin, prenatal vitamin, or folic acid supplement) every day of the month before pregnancy, 2) normal weight (prepregnancy body mass index 18.5–24.9 kg/m^2^), and 3) postpartum use of most or moderately effective contraceptive method (sterilization, implant, intrauterine device, or hormonal method [injectable, pill, patch, ring]); the remainder indicate health risks: 4) heavy alcohol use (8 or more drinks in an average week) during the 3 months before pregnancy, 5) cigarette use during the 3 months before pregnancy, 6) depression during the 3 months before pregnancy, 7) diabetes during the 3 months before pregnancy, 8) hypertension during the 3 months before pregnancy, and 9) unwanted pregnancy (defined as not wanting to be pregnant then or at any time in the future when thinking about how they felt just before they got pregnant) (2). The data for each indicator were self-reported by OPAS respondents, except for weight which was derived from the birth certificate. We did not assess physical activity, as this is not asked by OPAS. We also examined weighted percentages and 95% CIs of selected characteristics by county type (age; race and ethnicity; education; marital status; prenatal care initiation; Special Supplemental Nutrition Program for Women, Infants, and Children [WIC] support during pregnancy; health insurance at delivery), which are typically used when describing preconception health. We also assessed questions that were newly added to OPAS to examine potential inequities related to food security and neighborhood safety. We used Rao-Scott χ^2^ tests to assess for differences across county types and STATA 17.0 (StataCorp LLP) to account for the complex survey design for all analyses. Alpha was set a priori at .05. OPAS defines unreliable estimates when the denominator is less than 60 or the relative standard error is 30% or higher. The Ohio Department of Health Institutional Review Board deemed the analysis exempt.

## Results

During 2019–2021, 14,377 women completed OPAS (Table 1). A higher percentage of women living in rural non-Appalachian or Appalachian counties were younger than 25 years (28.8% and 39.9%, respectively) or uninsured (8.9% and 9.8%, respectively) as compared with metropolitan or suburban women. A higher percentage of women living in metropolitan or Appalachian counties reported Medicaid coverage (42.8% and 43.6%, respectively), support from WIC during pregnancy (30.7% and 33.8%, respectively), or eating less due to lack of money (9.3% and 9.8%, respectively) as compared with rural non-Appalachian or suburban women. A higher percentage of women living in Appalachian counties reported less than a high school education (20.7%) or later prenatal care initiation (16.9%) as compared with rural non-Appalachian, metropolitan, or suburban women. More racial and ethnic diversity was found among women living in metropolitan counties (57.4% white, non-Hispanic) as compared with other county types. A higher percentage of women living in metropolitan counties (11.7%) reported feeling unsafe in their neighborhood as compared with other county types.

**Table 1:** Characteristics of Ohio Women With a Live Birth, by County Type, Ohio Pregnancy Assessment Survey, 2019–2021^a^

Characteristic	All Ohio (N = 14,377)	Metropolitan (n = 11,555)	Appalachian (n = 808)	Rural, non-Appalachian (n = 872)	Suburban (n = 1,142)
	% (95% CI)				
**Age, y**					
<25	25.3 (24.1–26.5)	22.7 (21.6–23.9)	39.9 (35.5–44.4)	28.8 (24.8–33.1)	20.6 (17.3–24.3)
25–34	59.3 (58.1–60.6)	60.1 (58.8–61.3)	52.2 (47.7–56.6)	58.1 (53.8–62.3)	63.3 (59.5–67.0)
≥35	15.3 (14.5–16.1)	17.2 (16.3–18.1)	8.0 (6.2–10.3)	13.1 (10.7–16.0)	16.1 (13.7–18.7)
**Race and ethnicity**					
White, non-Hispanic	70.2 (69.3–71.3)	57.4 (56.2–58.6)	90.4 (87.1–93.0)	89.4 (86.3–91.8)	85.1 (82.0–87.8)
Black, non-Hispanic	16.6 (15.9–17.4)	26.1 (25.1–27.2)	3.1 (1.7–5.7)^b^	1.7 (0.8–3.7)^b^	5.2 (3.5–7.5)
Hispanic	6.1 (5.5–6.7)	7.5 (6.8–8.2)	3.3 (1.9–5.6)	4.5 (2.9–6.9)	4.6 (3.2–6.5)
Another race^c^	7.0 (6.4–7.6)	9.0 (8.3–9.8)	3.1 (1.8–5.3)	4.4 (3.0–6.4)	5.1 (3.7–7.2)
**Education, y**					
<12	10.7 (9.8–11.5)	9.5 (8.7–10.3)	20.7 (17.3–24.6)	9.6 (7.3–12.3)	8.2 (6.1–11.0)
12	26.5 (25.4–27.7)	25.5 (24.3–26.7)	36.1 (31.9–40.6)	29.3 (25.5–33.5)	20.5 (17.3–24.1)
>12	62.8 (61.5–64.0)	65.1 (63.8–66.3)	43.1 (38.8–47.5)	61.1 (56.8–65.2)	71.3 (67.4–74.9)
**Marital status**					
Married	55.0 (53.8–56.3)	52.0 (50.7–53.3)	53.1 (48.6–57.6)	59.3 (54.9–63.6)	64.0 (59.9–67.8)
Unmarried	45.0 (43.7–46.2)	48.0 (46.7–49.3)	46.9 (42.4–51.4)	40.7 (36.4–45.1)	36.0 (32.2–40.1)
**Prenatal care initiation**					
1st Trimester	86.2 (85.3–87.1)	86.1 (85.2–87.0)	79.9 (76.0–83.3)	88.7 (85.7–91.1)	89.2 (86.2–91.6)
2nd or 3rd Trimester	11.3 (10.5–12.1)	11.1 (10.4–12.0)	16.9 (13.8–20.6)	8.8 (6.7–11.5)	9.5 (7.3–12.4)
No prenatal care	2.5 (2.1–2.9)	2.7 (2.3–3.3)	3.2 (1.8–5.4)	2.5 (1.4–4.4)	1.3 (0.7–2.4)^b^
**WIC support during pregnancy**					
Yes	28.4 (27.3–29.6)	30.7 (29.4–31.9)	33.8 (29.6–38.2)	24.3 (20.6–28.4)	19.7 (16.6–23.3)
No	71.6 (70.4–72.7)	69.3 (68.1–70.6)	66.2 (61.8–70.4)	75.7 (71.6–79.4)	80.3 (76.7–83.4)
**Health insurance at delivery**					
Medicaid	38.4 (37.2–39.6)	42.8 (41.5–44.1)	43.6 (39.2–48.1)	27.5 (23.6–31.7)	27.4 (23.9–31.3)
Private	54.3 (53.1–55.6)	52.7 (51.4–53.9)	40.6 (36.4–45.0)	60.8 (56.5–64.9)	65.6 (61.7–69.3)
Other insurance	2.7 (2.3–3.2)	1.9 (1.6–2.3)	6.0 (4.2–8.5)	2.8 (1.7–4.8)	3.1 (2.1–4.6)
Uninsured	4.6 (4.0–5.1)	2.6 (2.2–3.1)	9.8 (7.5–12.6)	8.9 (6.8–11.6)	3.9 (2.6–5.9)
**Ate less due to lack of money** d					
Yes	8.5 (7.6–9.4)	9.3 (8.4–10.3)	9.8 (6.9–13.8)	6.4 (4.3–9.5)	5.9 (3.7–9.3)
No	91.5 (90.6–92.4)	90.7 (89.7–91.6)	90.2 (86.2–93.1)	93.6 (90.5–95.7)	94.1 (90.7–96.3)
**Felt unsafe in neighborhood** d					
Never	73.8 (72.5–75.1)	69.0 (67.6–70.3)	77.4 (72.3–81.9)	82.1 (77.7–85.8)	81.7 (77.5–85.3)
Rarely	16.9 (15.8–18.0)	19.3 (18.2–20.5)	15.8 (12.1–20.4)	12.2 (9.1–16.1)	13.1 (10.1–16.7)
Always/often/sometimes	9.2 (8.4–10.1)	11.7 (10.8–12.8)	6.7 (4.3–10.4)	5.7 (3.6–8.7)	5.2 (3.3–8.2)

Abbreviation: WIC, Special Supplemental Nutrition Program for Women, Infants, and Children.

a All indicators were self-reported by respondents.

b Interpret with caution; relative standard error is between 30% and 40%.

c Another race category includes those who identify as American Indian, Chinese, Japanese, Other Asian, Filipino, Hawaiian, other non-White, and mixed race. Insufficient data were available to present stable estimates for other racial groups. Sample sizes for the nonmetro county groupings ranged from 0 to 28.

d Variables were not asked in the 2019 survey; percentages represent only data from the 2020–2021 Ohio Pregnancy Assessment Survey. Respondents were asked whether they had these experiences during the 12 months before delivery.

We found significant differences among county types for 6 of the 9 preconception health indicators (Table 2). Thirty percent of women living in Appalachian counties reported taking folic acid every day in the month before pregnancy, the lowest among the geographic areas. Prevalence of cigarette use before pregnancy was highest among women living in Appalachian (23.1%) and rural non-Appalachian (21.6%) counties. Women living in Appalachian counties reported the highest prevalence of depression before pregnancy (27.8%). Rates of diabetes (1.3%) or hypertension (3.5%) before pregnancy, as well as unwanted pregnancy (21.1%), were lowest among women living in suburban areas.

**Table 2:** Preconception Health Indicators Among Ohio Women With a Live Birth, Ohio Pregnancy Assessment Survey, 2019–2021^a^

Indicator	All Ohio	Metropolitan	Appalachian	Rural, non-Appalachian	Suburban	*P* value^b^
	% (95% CI)					
Folic acid intake^c^	37.5 (36.3–38.6)	38.0 (36.8–39.3)	30.0 (26.1–34.0)	36.8 (32.8–40.8)	41.8 (38.1–45.5)	<.001
Normal weight^d^	40.6 (39.4–41.9)	40.1 (39.0–41.4)	39.8 (35.4–44.2)	40.2 (36.0–44.4)	43.3 (39.6–47.1)	.46
Most or moderately effective contraception use postpartum^e^	50.7 (49.5–52.0)	51.2 (49.9–52.4)	48.5 (44.1–53.0)	51.8 (47.5–56.1)	50.1 (46.3–53.9)	.65
Heavy alcohol use^f^	2.8 (2.4–3.3)	3.0 (2.5–3.4)	3.9 (2.1–5.7)	2.2^g^ (0.9–3.5)	2.1 (1.1–3.2)	.24
Cigarette use^h^	17.9 (16.9–19.0)	15.9 (14.8–16.9)	23.1 (19.2–26.9)	21.6 (17.9–25.4)	18.4 (15.2–21.6)	<.001
Depression^i^	22.0 (20.9–23.1)	20.6 (19.5–21.7)	27.8 (23.7–31.8)	23.2 (19.5–26.9)	21.4 (18.1–24.7)	.005
Type 1 or type 2 diabetes^i^	2.8 (2.4–3.2)	3.1 (2.7–3.6)	3.7 (1.9–5.5)	2.6 (1.4–3.9)	1.3^g^ (0.4–2.2)	.04
Hypertension^i^	5.6 (5.1–6.2)	6.3 (5.7–7.0)	5.1 (3.2–7.0)	5.6 (3.7–7.6)	3.5 (2.2–4.8)	.03
Unwanted pregnancy^j^	25.3 (24.2–26.4)	25.8 (24.6–27.0)	28.8 (24.6–33.0)	25.1 (21.2–29.0)	21.1 (17.8–24.4)	.03

a All indicators are self-reported by survey respondents 2 to 6 months after delivery except for normal weight, which is derived from the birth certificate. Missingness ranged from 0% (normal weight) to 0.8% (most or moderately effective contraception; unwanted pregnancy). Percentages are weighted.

b Rao-Scott χ^2^ test comparing county types (metropolitan, Appalachian, rural non-Appalachian, and suburban).

c Defined as taking a multivitamin, prenatal vitamin, or a folic acid supplement every day of the month before pregnancy.

d Defined as a prepregnancy body mass index 18.5–24.9 kg/m^2^, as derived from height and weight listed on the birth certificate.

e Defined as reporting current use of 1 of the following methods to keep from getting pregnant: sterilization, implant, intrauterine device, injectable, pill, patch, or ring.

f Defined as consuming 8 or more drinks in an average week during the 3 months before pregnancy.

g Interpret with caution; relative standard error is 30%–40%.

h Defined as any cigarette smoking in the 3 months before pregnancy.

i During the 3 months before pregnancy.

j Defined as not wanting to be pregnant then or at any time in the future when thinking about how they felt just before they got pregnant.

## Discussion

Geographic differences in preconception health exist in Ohio by county type, potentially affecting women’s health and pregnancy outcomes. These results confirm previously published findings of geographic differences in preconception health in the United States (9–12). However, to our knowledge, this is the first peer-reviewed publication examining preconception health indicators by county type in Ohio. Previous literature indicates that these differences may be due to health disparities driven by challenges related to social determinants of health (eg, educational attainment, food insecurity) and access to care (eg, health insurance) (11,13). Examining preconception health indicators by region prevents masking variation that may not be apparent in statewide estimates. By examining 3 years of data, we had sufficient sample size to examine differences among the geographic locations that can inform local public health practice.

The main limitation of this study is that results are applicable only to Ohio. Strengths include generalizability of results to geographically diverse Ohio women with a live birth. Although these results are specific to Ohio, these data add to the limited literature regarding preconception health indicators in rural and Appalachian populations (9,10,12).

Improving preconception health is central to optimizing pregnancy outcomes and women’s health (1–5). Preconception health status affects the health of future generations; for example, morbidity or modifiable preconception health behaviors may influence fetal development or lead to complications during pregnancy such as premature birth, which in turn may result in infant mortality or long-term complications for offspring (4,5). Improving preconception health can also mitigate the development of chronic disease among women during and after their reproductive years, improving overall well-being and quality of life (3,11). Differences in preconception health by residence location show the need for a more focused approach to implement local interventions in Ohio to achieve health equity, particularly in regions with persistent health disparities. States with distinct geographic regions may consider regional analyses to better inform state efforts.
